# A prevalent mutation with founder effect in Spanish Recessive Dystrophic Epidermolysis Bullosa families

**DOI:** 10.1186/1471-2350-11-139

**Published:** 2010-09-29

**Authors:** Natividad Cuadrado-Corrales, Carolina Sánchez-Jimeno, Marta García, María-José Escámez, Nuria Illera, Ángela Hernández-Martín, María-José Trujillo-Tiebas, Carmen Ayuso, Marcela Del Rio

**Affiliations:** 1 Basic Research Department, Epithelial Biomedicine Division, Regenerative Medicine Unit, CIEMAT, Madrid, Spain; 2 Centro de Investigaciones Biomédicas en Red de Enfermedades Raras (CIBERER- U714), Madrid, Spain; 3 Department of Dermatology, Hospital Niño Jesús, Madrid, Spain; 4 Department of Genetics, Fundación Jiménez Díaz, Madrid, Spain; 5 Centro de Investigaciones Biomédicas en Red de Enfermedades Raras (CIBERER- U704), Madrid, Spain

## Abstract

**Background:**

Recessive Dystrophic Epidermolysis Bullosa (RDEB) is a genodermatosis caused by more than 500 different mutations in the *COL7A1 *gene and characterized by blistering of the skin following a minimal friction or mechanical trauma.

The identification of a cluster of RDEB pedigrees carrying the c.6527insC mutation in a specific area raises the question of the origin of this mutation from a common ancestor or as a result of a hotspot mutation. The aim of this study was to investigate the origin of the c.6527insC mutation.

**Methods:**

Haplotypes were constructed by genotyping nine single nucleotides polymorphisms (SNPs) throughout the *COL7A1 *gene. Haplotypes were determined in RDEB patients and control samples, both of Spanish origin.

**Results:**

Sixteen different haplotypes were identified in our study. A single haplotype cosegregated with the c.6527insC mutation.

**Conclusion:**

Haplotype analysis showed that all alleles carrying the c.6527insC mutation shared the same haplotype cosegregating with this mutation (***CCGCTCAAA_6527insC***), thus suggesting the presence of a common ancestor.

## Background

**Figure 1:**
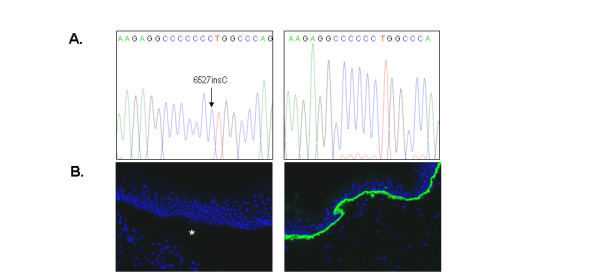
**Molecular and microscopic characterization of RDEB patients that are homozygotes for c.6527insC in exon 80**. (A) Identification of the c.6527insC mutation by direct DNA sequence analysis. The chromatogram shows the pattern of 7 C peaks typically found in these patients (left) when compared with healthy controls (right). (B) Indirect immunofluorescent staining with LH7.2 monoclonal antibody (NC-1 domain of type VII collagen). The control section shows a continuous staining along the intact dermal-epidermal junction (right), while the section from the patient is negative to the staining and show a dermal-epidermal cleavage (*).

**Figure 2:**
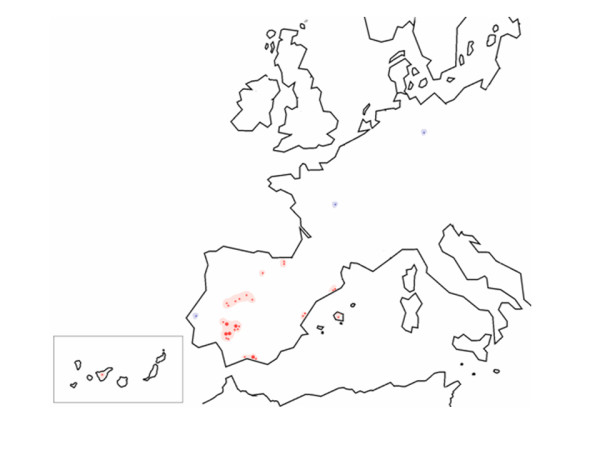
**Geographic location of alleles carrying the c.6527insC mutation in RDEB patients (red circles)**. Circle size is proportional to the number of CCGCTCAAA_6527insC alleles. The mutation follows a south-north spatial gradient in the Iberian Peninsula, from Andalusia to Northern Spain. Note that only three alleles that carry the c.6527insC mutation occur in the rest of Europe (blue circles, France, Germany and Portugal)

Dystrophic Epidermolysis Bullosa (DEB) is a rare disease, characterized by trauma induced-blistering and scarring [1]. This genodermatosis is a rare autosomal dominant (DDEB [MIM#131750, #131800]) or recessive (RDEB [MIM#226600]) disorder caused by mutations in *COL7A1 *gene [MIM*120120], encoding type VII collagen (protein component of anchoring fibrils) [2]. *COL7A1 *gene is an unusually complex gene with 118 exons. It has the second largest number of exons of all genes described to date [3]. So far, more than 500 mutations have been described in the *COL7A1 *gene http://www.hgmd.cf.ac.uk[4]. Although *COL7A1 *genetic database indicates that most of the DEB mutations are family specific, with few recurrent mutations, in the Spanish cohort, a high recurrence of the c.6527insC pathogenic mutation has recently been reported by our group [5]. Accounting for 46.3% of alleles this is a level of recurrence for a single genetic defect hardly ever found for the *COL7A1 *gene [5,6]. The c.6527insC mutation creates a premature codon termination (PTC), leading to nonsense mediated decay (NMD) that manifests as a complete absence of collagen protein (Fig. 1). This insertion mutation was detected mainly in families native of the southwest of the Iberian Peninsula (Fig. 2). The overrepresentation of a single mutation and the geographic clustering of the c.6527insC pedigrees indicate at least one founder effect or a mutational hotspot. We investigated the putative founder effect of the c.6527insC mutation through the construction of SNP haplotypes throughout the *COL7A1 *gene. A rare single haplotype in the Spanish population was present in all patients and relatives who carried the c.6527insC mutation, supporting the hypothesis that all chromosomes carrying the c.6527insC mutation arise from a single founder effect.

## Methods

### Subjects

A total of 49 DEB patients were included in this study. Patient written informed consent was obtained in agreement with the collaborative centers where biopsies and blood/DNA samples were obtained. The Ethics Committee of Fundación Jimenez Díaz (Madrid, Spain) evaluated and approved this research work, stating that the project adheres to the Helsinki Guidelines and further reviews (Edinburgh, 2000; http://www.wma.net).

Eleven RDEB patients carried the c.6527insC mutation in both alleles. Sixteen RDEB patients were heterozygote, exhibited the c.6527insC mutation on one allele and contained different mutations on the other allele. Twenty two DEB patients carried other mutations of the *COL7A1 *gene (Additional file 1, Table S1). In addition, 93 non-affected individuals from the general population of Spain were incorporated to this study as a reference group. Informed consent was obtained from all subjects included in the study. Putative geographic origin of pedigrees was empirically established based on proof that individuals carrying this mutation stem from ancestors who have lived in the same geographic area for at least 3 generations.

### SNPs selection and haplotyping assay

**Figure 3:**
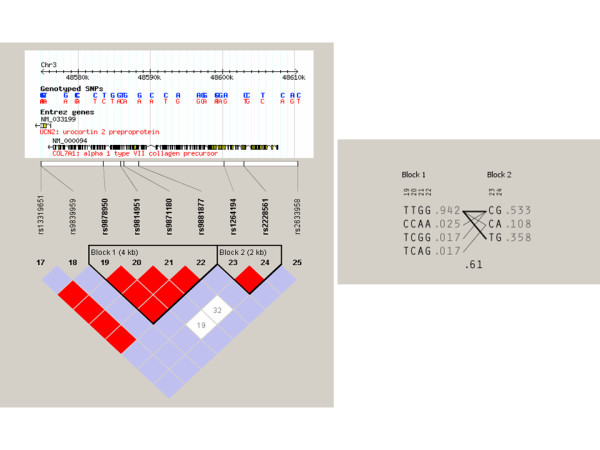
**Haplotype structure of the COL7A1 gene locus in Europeans**. (A) Structure of the COL7A1 gene with positions of SNPs by the Hap Map project. Haploview-generated linkage disequilibrium (LD) patterns of the COL7A1 gene and predicted block structure in Caucasian control subjects. Rate (D') of LD is represented by different colors (highest rate of LD in red, lower LD in purple, and white for no LD). (B) Potential haplotypes in the European population for six selected SNPs that have been genotyped in our study.

We used the HapMap data http://www.hapmap.org to select informative SNPs from the *COL7A1 *gene. Using the Haploview program, two blocks of linkage disequilibrium (LD) are located throughout the *COL7A1 *gene (Fig. 3). Two SNPs (rs2228561 and rs1264194) from block 1 and five SNPs (rs9881877, rs9871180, rs9814951, rs9878950 and rs2532848) from block 2 were analyzed to determine common haplotypes according to the Hap-Map data. We also included in the analysis two novel polymorphisms, NM-000094.3:c.25215C >T (intron 75) and NM-000094.3:c.11639C >T (intron 19) recognized in the Spanish population.

**Figure 4:**
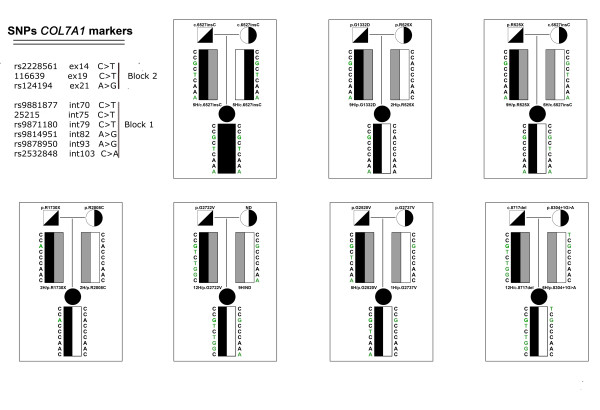
**Some pedigrees of Spanish cases of DEB are shown as an example**. The existence of complete two-generation pedigrees in DEB patients allowed construction of different haplotypes throughout the COL7A1 gene. The haplotypes are boxed. Symbols are encoded as follows: black, DEB patients; white and black, carrying recessive mutations

Haplotypes for chromosomes harboring the c.6527insC mutation and other mutations were determined by genotyping SNPs for DEB patients and their parents (Fig. 4).

The genotyping of SNPs was performed with Snapshot assay kits (Applied Biosystems Inc.). Amplicons were purified by Exo-SAP treatment (ExoSAP-IT, USB). Three μl aliquots of the purified amplification products were added to a 5 μl Snapshot Multiplex Ready reaction mixture containing 0.4 pmol of each Snapshot oligonucleotide primers (Additional file 1, Table S2). Primer extension reactions were carried out over 25 cycles of 96°C for 10 sec, 50°C for 5 sec, and 60°C for 30 sec. Reaction products were treated with 1 U of SAP at 37°C for 1 h and 72°C for 15 min to dephosphorylate unincorporated fluorescent ddNTPs. The Snapshot reactions were resolved on an ABI Prism 3730 (Applied Biosystems). Results were analyzed using Peak Scanner™ Software v1.0 (Applied Biosystems).

### Statistical analysis

We evaluated the Hardy-Weinberg Equilibrium (HWE) distributions, in a control sample from the general population of Spain, for the SNPs involved in our study.

## Results

We identified 5 novel SNPs in the region corresponding to *COL7A1 *gene, NM-000094.3:c.11639C>T, 24558C>T, 25215C>T, 29056C>T, 31427C>A. The distribution of all SNPs in the control Spanish population did not deviate from HWE (?2-test; p > 0.05) (Additional file 1, Table S3). The NM-000094.3:c.11639C>T and 25215C>T were considered for the construction of haplotypes.

The existence of complete two-generation pedigrees in all our patients allowed the construction of different haplotypes formed by nine SNPs. Considering the two blocks of linkage LD located throughout the *COL7A1 *gene, we determined twelve haplotypes in DEB patients (from H1 haplotype to H7 haplotype, H9, H12, H14, H15 and H16 haplotypes) and thirteen haplotypes in the Spanish population (from H1 to H13). The overall distribution of the estimated haplotypes was significantly different between healthy controls and patients (see Table 1 and Table 2).

**Table 1:** The total number of haplotypes described in the Spanish population

	Block 2			Block 1						
			
Haplotypes	rs2228561	11639 C>T	rs1264194	rs9881877	25215C>T	rs9871180	rs9814951	rs9878950	rs2532848	Haplotype frequency %
H1	C	C	G	C	C	C	A	A	C	33.7
H2	C	C	A	C	C	C	A	A	C	17.44
H3	C	C	A	C	C	C	G	G	C	10.46
H4	C	C	G	C	C	C	A	G	C	5.81
H5	C	C	G	C	T	C	A	A	A	5.81
H6	T	C	G	C	C	C	A	A	C	4.65
H7	C	C	G	T	C	C	A	A	C	4.65
H8	C	C	G	C	C	C	G	G	C	4.65
H9	C	C	G	C	C	C	A	A	A	3.49
H10	C	C	G	C	T	C	A	A	C	3.49
H11	C	-	A	C	C	C	A	G	C	3.49
H12	C	C	G	T	C	T	G	G	C	1.16
H13	C	-	A	C	T	C	A	A	C	1.16
H14	C	T	G	C	T	C	A	A	A	0
H15	C	T	G	C	T	C	A	G	A	0
H16	C	T	G	C	C	C	A	A	C	0

**Table 2:** The total number of haplotypes described in DEB Spanish patients

		**Block 2**			**Block 1**					
			
**Haplotypes**	**Mutation COL7A1 gene**	**rs2228561**	**11639 C>T**	**rs1264194**	**rs9881877**	**25215C>T**	**rs9871180**	**rs9814951**	**rs9878950**	**rs2532848**
		
H1	p.Y112X (1), p.R185X (2), p.G1318R (1), p.G1791E (1), p.R1814C (1), p.G2061V (1), p.G2061V (1), p.R2424W (1), p.G2587 D (1), p.R2622W(1), p.2737V (1), c.267-3C>G (2), c.4401+1G>A(1), c.7420A>G (1), c.7930-1G>C (3), c.7930+2T>C, c.7756insC.	C	C	G	C	C	C	A	A	C
										
H2	p.R525X (1), p.R1730X (1), p.R2063W (1), c.7104+5G>A (1), c.5576delAA (1), c.325insGC (2),	C	C	A	C	C	C	A	A	C
										
H3	p.G2434R (1), p.R2808C (1)	C	C	A	C	C	C	G	G	C
										
H4	c.58del13 (1)	C	C	G	C	C	C	A	G	C
										
**H5**	**c.6527insC (38), p.G2520V (1)**	**C**	**C**	**G**	**C**	**T**	**C**	**A**	**A**	**A**
										
H6	p.Y1098X (2), c.2781InsACGAC (1), c.8304+1G>A (1)	T	C	G	C	C	C	A	A	C
										
H7	p.G2366 D (1), p.G2114 D (1)	C	C	G	T	C	C	A	A	C
										
H8	-	C	C	G	C	C	C	G	G	C
										
H9	p.G1332 D (1), c.R525X (1), ND(1)	C	C	G	C	C	C	A	A	A
										
H10	-	C	C	G	C	T	C	A	A	C
										
H11	-	C	-	A	C	C	C	A	G	C
										
H12	p.G2722V, c.8717delC (1), ND (1).	C	C	G	T	C	T	G	G	C
										
H13	-	C	T	A	C	T	C	A	A	C
										
H14	c.5131nsCTCAC (2), c.3277-1G>C (1)	C	T	G	C	T	C	A	A	A
										
H15	ND (2)	C	T	G	C	T	C	A	G	A
										
H16	c.7929+2T>C (1)	C	T	G	C	C	C	A	A	C

The total number of the alleles (n = 38) that carry the c.6527insC mutation cosegregated exclusively with H5 haplotype (CCGCTCAAA), namely all alleles that carry the c.6527insC mutation were ***CCGCTCAAA_6527insC***. On the other hand, the frequency of H5 haplotype in the Spanish population was 5.81%. Consequently, the c.6527insC mutation in the Spanish cluster is a result of a single mutational event, and the affected pedigrees must descend from one genetic founder who exhibited the H5 haplotype. Out of 27 pedigrees bearing at least one CCGCTCAAA_6527insC allele, 26 stemmed from ancestors who had been living in the southern half of the Iberian Peninsula for at least 3 generations. The other lineage bearing the CCGCTCAAA_6527insC allele comes from ancestors who had been living in Northern Spain for over 3 generations. In addition, we analyzed a Portuguese pedigree carrier of the CCGCTCAAA_6527insC allele. These results reveal that the c.6527insC mutation is not the result of separate mutations occurring independently in different individuals, but is the result of a one-time mutation occurring in a common ancestor of all the families. Furthermore, we also found a pedigree where the chromosome carrying the mutation cosegregated with G2520V H5 haplotype. The other forty recessive pathogenic mutations cosegregate with other fifteen different haplotypes (Table 2).

## Discussion

High carrier rates are usually attributed to a founder effect in a population and are usually evidenced by conservation of haplotypes with directly associated markers [7]. We constructed fifteen different intragenic haplotypes throughout the *COL7A1 *gene to explore the origin of the alleles carrying the c.6527insC mutation and other mutations described in our DEB patients. All alleles carrying the c.6527insC mutation were CCGCTCAAA_6527insC, indicating a common origin. This hypothesis is supported by the diversity of haplotypes throughout the *COL7A1 *gene. Moreover, H5 haplotype is rare in the control population (5.81%).

The c.6527insC mutation is found at a high prevalence among patients from the southern half of the Iberian Peninsula. This mutation has previously been found in one patient in France [8] and in another in Germany [9]. However, a Spanish predecessor of those patients cannot be excluded, taking into account the large Spanish emigration to France that occurred after the Spanish civil war (1936-1939) and to Germany in the early 1960 s due to economic hard-ship.

The overall distribution of the estimated haplotypes was significantly different between patients and healthy controls. The absence of recurrence in the other mutations did not allow us to distinguish whether a single haplotype is associated with a mutation, or conversely, if one mutation is associated with general haplotypes suggesting multiple origins. The H1 and H2 haplotypes identified as cosegregating with other mutations are common in the background population. This finding is neither surprising nor unexpected. In terms of elementary probability, the probability of occurrence of a pathogenic mutation in a chromosome harboring a relatively common haplotype in the population is obviously high.

We found a single mutation, p.R525X, which cosegregated with two different haplotypes (H2 haplotype and H9 haplotype). Moreover this nonsense mutation has been described previously in other DEB patient cohorts [10]. This could indicate the presence of a mutational hotspot in codon 525 of *COL7A1 *gene. Codon 525 contains a CpG dinucleotide, which is the known site of DNA modification by cytosine methylation.

## Conclusion

In summary, the conservation of a single haplotype surrounding the c.6527insC mutation suggested that this allele has a single origin. The finding of a founder effect in a highly recurrent mutation in a rare disease characterized by intrafamilial mutations is essential for the implementation of protocols for genetic diagnosis, for genetic counselling of affected pedigrees and is fundamental to search for new therapies.

## Competing interests

The authors declare that they have no competing interests.

## Authors' contributions

NCC drafted the manuscript and conceived and designed the study concept. MDR coordinated the analysis and interpretation of data and was responsible for final approval of the article. CSJ, MG and NI performed and assembly the molecular data, MJE contributed to the collection and assembly of clinical data and the statistical analysis. AHM performed the dermatological examinations. MJTT and CA provided critical revision of the manuscript.

All authors read and approved the final manuscript.

## Pre-publication history

The pre-publication history for this paper can be accessed here:

http://www.biomedcentral.com/1471-2350/11/139/prepub

## Supplementary Material

Aditional file 1 **Supplementary tables**. Table S1. Summary of DEB Spanish patients included in this study. Table S2. Primer sequences used for Analysis of SNPs throughout the *COL7A1 *gene and primers used for SNaPshot. Table S3. Novel *COL7A1 *SNPs recognized in the present study. Click here for file
