# Supplementary material for: A prevalent mutation with founder effect in Spanish Recessive Dystrophic Epidermolysis Bullosa families
Source: BMC Med Genet. 2010 Sep 29;11:139. doi: 10.1186/1471-2350-11-139 (PMC2957067; doi:10.1186/1471-2350-11-139)
Supplement: Aditional file 1 — Supplementary tables. Table S1. Summary of DEB Spanish patients included in this study. Table S2. Primer sequences used for Analysis of SNPs throughout the COL7A1 gene and primers used for SNaPshot. Table S3. Novel COL7A1 SNPs recognized in the present study. [file 1471-2350-11-139-S1.DOC]

**Table S1**. Summary of DEB Spanish patients included in this study.

| **Nr. of patients** | **Clinical diagnosis** | ***COL7A1* gene mutation** | |
| --- | --- | --- | --- |
|  |  | **Mutation 1**  **Designation** | **Mutation 2**  **Designation** |
| 11 patients | RDEB-sev gen | c.6527insC | c.6527insC |
| 1 | RDEB-sev gen | c.6527insC | p.Y112X |
| 1 | RDEB-sev gen | c.6527insC | ND |
| 1 | RDEB-sev gen | c.6527insC | c.58del13 |
| 1 | RDEB-sev gen | c.6527insC | ND |
| 1 | RDEB-sev gen | c.6527insC | c.5131insCTCAC |
| 1 | RDEB-sev gen | c.6527insC | c.5131insCTCAC |
| 1 | RDEB-sev gen | c.6527insC | p.G2366D |
| 1 | RDEB-sev gen | c.6527insC | p.G2587D |
| 1 | RDEB-sev gen | c.6527insC | ND |
| 1 | RDEB-sev gen | c.6527insC | p.R525X |
| 1 | RDEB-sev gen | c.6527insC | c.7929+2T>C |
| 1 | RDEB-O | c.6527insC | p.G2434R |
| 1 | RDEB-O | c.6527insC | p.G1383R |
| 1 | RDEB-O | c.6527insC | c.7104+5G>A |
| 1 | RDEB-ac | c.6527insC | p.R1814C |
| 1 | RDEB-Pt | c.6527insC | p.G2114D |
| 1 | RDEB-sev gen | c.7756 insC | ND |
| 1 | RDEB-sev gen | c.267-3C>G | c.267-3C>G |
| 2 | RDEB-sev gen | c.325insGC | c.3277-1G>C |
| 1 | RDEB-sev gen | p.Y1098X | p.Y1098X |
| 1 | RDEB-sev gen | p.G2722V | ND |
| 1 | RDEB-I | p.R185X | p.R2622W |
| 1 | RDEB-O | c.7420A>G | c.5576delAA |
| 1 | RDEB-O | p.R2808C | p.R1730X |
| 1 | RDEB-O | p.R185X | c.7320+2T>C |
| 1 | RDEB-O | p.G1338R | c.4401+1G>A |
| 1 | RDEB-O | p.R525X | p.G1332D |
| 1 | RDEB-O | c.7930-1G>C | c.7930-1G>C |
| 1 | RDEB-O | p.R2063W | ND |
| 1 | RDEB-Pt | G2520V | G2737V |
| 1 | RDEB-Pt | c.G2221A | c.7930-1G>C |
| 1 | RDEB-ac | c.8717delC | c.8304+1G>A |
| 1 | RDEB-Ac | c.2781InsACGAC | p.R2424W |
| 1 | RDEB-sev gen | ND | ND |
| 1 | DEB-Pr | p.G1791E | WT |
| 2 | DDEB-gen | p.G2061V | WT |

**Table S2**. Primer sequences used for Analysis of SNPs throughout the *COL7A1* gene and primers used for SNaPshot

| **SNP** | **Base substitution** | **Sense/Antisense primers (5´-3´)** | **Amplicon length (bp)** |
| --- | --- | --- | --- |
| rs2228561 | C>T | Forward: TGAGTACTGCAGGAGGCTTG  Reverse: TGAGGTCAGAGGGAAATGCT  SNaPshot: TCTGTCTGTAACTCCTAGAGCC | 314 |
| rs1264194 | A>G | Forward: AACCCAGTTAACAGAGCCAG  Reverse: GGAGGAGTCACTCAGAGTCG  SNaPshot: (T)_7_AGGCTGAGTGTCCTAGGGCCA | 330 |
| rs9881877 | C>T | Forward: TGTAGCCCACACTCAAGGGAA  Reverse: TACCCCCAGCCCCTAAACAA  SNaPshot: (T)_14_GTGCTTTCCTGAGGCCGTGC | 272 |
| 25215C>T | C>T | Forward: CCAGGAGAGTGAGGGAAGAG  Reverse: TAGGGTCAGAAATTCCAGGG  SNaPshot: (T)_25_CACCCCATCCCTGCCTTAGTC | 370 |
| rs9871180 | C>T | Forward: ATGTGGATGTGTGTGTGCAG  Reverse: AAGGTTCTTGGGTACTCACCA  SNaPshot:(T)_32_GGAAATTGACCCCCAAGGAAAAAGCC | 273 |
| rs9814951 | A>G | Forward: CCTTCTCTGTGTTAATCCCTG  Reverse: CTCATTGCAGGAGATGACAGC  SNaPshot: (T)_18_GGTGAGTCTAGGTGTGTGGATA | 202 |
| rs9878950 | A>G | Forward: CCTGAGCTTGATCCGATGC  Reverse: GGGAATGGTCAATGCAGGAC  SNaPshot: (T)_31_AATGCCCCATCACCAGTTGTA | 191 |
| rs2532848 | C>A | Forward: CGGGCTCGTTGTATTCTAAG  Reverse: CAAAAGCTACCACACTGGT  SNaPshot: (T)_44_GATCTGTGTGTGGCCTGGGC | 514 |

**Table S3**. Novel *COL7A1* SNPs recognized in the present study.

| **Name** | **Localization *COL7A1* gene** | **Minor allelic frequency**  **(control sample)** | **Genotype frequency**  **(control sample)** | **χ^2^ test (*p*)** |
| --- | --- | --- | --- | --- |
| C>T 11639 | intron 13 | 0.05 | CC/CT/TT  80/8/0  0.909/0.099/0.0 | 0.65 |
| C>T 24558 | exon 72 | 0.01 | CC/CT/TT  42/1/0  0.976/0.023/0.0 | 0.94 |
| C>T 25215 | intron 75 | 0.22 | CC/CT/TT  49/36/1  0.569/0.418/0.011 | 0.045 |
| C>T 29056 | exon 92 | 0.04 | CC/CT/TT  48/4/0  0.923/0.076/0.0 | 0.77 |

Genotypic and allelic counts, frequencies, and exact test for Hardy-Weinberg Equilibrium p-value.

The distribution of all SNPs in the control Spanish population did not deviate from HWE.

(χ2-test; *p*>0.05)
